# *Bacillus megaterium* Strain CDK25, a Novel Plant Growth Promoting Bacterium Enhances Proximate Chemical and Nutritional Composition of *Capsicum annuum* L

**DOI:** 10.3389/fpls.2020.01147

**Published:** 2020-07-30

**Authors:** Kalpana Bhatt, Dinesh Kumar Maheshwari

**Affiliations:** Department of Botany and Microbiology, Gurukula Kangri University, Haridwar, India

**Keywords:** cow dung bacteria, plant growth-promotion, *Bacillus megaterium*, proximate content, nutrient content, *Capsicum annuum* L.

## Abstract

The present study aimed to scrutinize the effect of different cow dung bacterial treatments on the nutritional value of *Capsicum annuum* L. Among all treatments, seeds inoculated with *Bacillus megaterium* (CDK25) showed significant enhancement in various proximate constituents *viz*., crude fiber (3.31%), crude protein (3.84%), and ash (2.53%) as compared to control. Likewise, significant increase in different nutrient contents *viz*., Ca (16.26 mg/100 g), Mg (17.37 mg/100 g), P (11.91 mg/100 g), K (0.47 mg/100 g), Fe (1.37 mg/100 g), and Zn (0.21 mg/100 g) was recorded over the control. Principal component analysis data depicts a positive correlation between different treatments with variables, validating enhancement in nutritional constituents by *B. megaterium* (CDK25) treatment. The study suggests the application of “*B. megaterium*” for achieving the persistent potential for augmenting and boosting up plant biological, functional, and nutritional assets, thereby enhancing the overall edible quality of *C. annuum* L. along with weathering of soil minerals.

## Introduction

*Capsicum annuum* L. (Chilli) is an essential crop of India having high agricultural, nutritional, commercial as well as medicinal value. It accounts for 25% of world production and is grown almost throughout the country (Nayaka et al., 2009). It is the third most crucial crop belonging to the *Solanaceae* family after potato and tomatoes. Besides, considered as a remunerative spice-crop of the Indian subcontinent occupying about 0.81 million ha (Raj and Christopher, 2009). Chilli is the maximum produced spice-crop bearing natural colors and antioxidant compounds, intake of which in adequate amounts prevents cancer and cardiovascular diseases (Brainley, 2000). Moreover, intended for coloring and flavoring food items, it provides essential nutrients and vitamins, too (Mussema, 2006). Its small amounts in the supplementary daily diet provide plenty of health benefits. It has various pharmacological properties *viz*., anti-inflammatory, antihemorrhoidal, antioxidant, antiobesity, antipyretic, analgesic, gastroprotective, besides providing relief from rhinitis sinusitis, diabetes, and arthritis. Its annual trade is around 17% of the total spice trade in the world and about 33% in India; this is because of its high monetary merit and consumption outlay (Ahmed et al., 2000). Owing to its various properties, farmers face more challenges of meeting growing demand. To counter the loss in productivity, farmers entirely hinge on the supplementation of chemical fertilizers. In India, the application of chemical fertilizers has tremendously increased 170 times over the last 50 years (Datta et al., 2011). Long term usage of chemicals has a toxic impact on the agricultural and aquatic environment. Not only does the deposition of chemical traces affect the soil profile, environment, and groundwater pollution, but the consumption of such chemical deposits in agriculture products also results in severe health issues (Kibblewhite et al., 2008; Singh et al., 2008).

Therefore, to overcome such a problem, it is substantially imperative to use the alternative eco-friendly approach. One of them is the bacterial inoculant practice, generally, the plant growth-promoting bacteria (PGPBs); offering ecological benefits to plant growth promotion besides maintaining soil eco-profile (Bashan and Holguin, 1998). PGPBs have direct and indirect plant growth-promoting metabolites that enhance active compounds of multifarious importance (Glick, 2012). PGPBs are capable of transforming insoluble fractions of soil nutrients (organic and inorganic) to soluble one by various processes *viz*., solubilization, chelation, mineralization, oxidation, or reduction, thus enhancing their accessibility for the plant (Shen et al., 2011). Cow dung is being practiced since ancient times, the addition of which increases soil mineral status along with enhanced plant growth. Cow dung consists of several minerals, fibers, and crude protein besides consisting beneficial microorganisms *viz*., bacilli, cocci, lactobacilli, fungus, and yeast (Muhammad and Amusa, 2003). Unfortunately, limited attention has been paid by researchers on cow dung bacteria in mediating soil nutrients’ cycle. *Bacillus subtilis* procured from cow dung, enhancing plant growth, oxidizing sulfur, and solubilizing phosphorus have been reported well (Swain and Ray, 2006). Likewise, Stefan et al. (2013) already reported *Bacillus* spp. for enhancing soil nutrients (macro and micro) along with their accessibility for the host plant. The effectiveness of these bacteria is mainly due to their incompetent rhizosphere, non-native nature, and other edaphic factors.

Various comparative studies on the estimation of the nutritive and chemical composition of different *C. annuum* varieties have been described earlier (Emmanuel-Ikpeme et al., 2014; Zou et al., 2015; Sharma et al., 2017). Till date, no such study using plant growth-promoting cow dung bacteria exploited the enhancing nutritional contents of *C. annuum* L. In this light, this research was framed with the objectives: (i) to assess the potential of cow dung bacteria possessing multiple growth promotion traits concerning vegetative and reproductive parameters of *C. annuum* L., (ii) to decipher the effect of potential bacterial treatment on proximate composition: moisture, ash, fat, crude protein, crude fiber, and carbohydrate, (iii) to evaluate the effect of bacterial treatment on nutrient contents: macro (calcium, magnesium, phosphorous, and potassium) and micro (zinc, iron, and copper) of *C. annuum* L., and (iv) to evaluate the statistical correlation between different bacterial treatments on proximate and nutrient constituents variables of *C. annuum* L.

## Materials and Methods

### Procurement of Bacterial Strains, Culture Conditions and Characterization of Plant Growth-Promoting (PGP) Assets

Two (CDK15 and CDK25) bacterial isolates were procured from the Dept. of Botany and Microbiology, Gurukula Kangri University, Haridwar (Uttarakhand), India. Both the isolates were obtained from our previous study (Bhatt and Maheshwari, 2019) and maintained in Luria-Bertani medium (LB) comprising the ingredients *viz*., 5 g yeast extract, 10.0g tryptone, 10.0 g NaCl L^−1^ respectively, at 30 ± 1°C. The isolates were characterized for various plant growth-promoting assets *viz*., phosphate solubilization, zinc solubilization, Indole-3-acetic acid production, siderophore production, and hydrogen cyanide production, of which, the phosphate solubilization potential was assessed qualitatively and quantitatively on Pikovskaya’s agar medium (Dubey and Maheshwari, 2011). The zinc solubilization efficiency was evaluated qualitatively and quantitatively on Bunt and Rovira medium (Bhatt and Maheshwari, 2020). Indole-3-acetic acid production was also assessed qualitatively as well as quantitatively by a colorimetric method (Rahman et al., 2010). The siderophore production was determined on Chrome-azurol S (CAS) medium (Schwyn and Neilands, 1987). Hydrogen cyanide production was estimated by the color development procedure (yellow-brown: light, moderate, or strong) as described by Kumar et al. (2012) (**Table 1**).

**Table 1:** Plant growth-promoting traits of selected bacterial isolates.

Isolates	Phosphate		Zinc		IAA (μg/ml)	SP	HCN
	SI (cm)	AS (mg/ml)	SI (cm)	AS (ppm)			
CDK15	3.46	264.04	3.5	14.0	11.6	+	+
CDK25	3.30	281.59	3.7	20.0	13.8	++	++

Where, + + (maximum), + (moderate). SI, Solubilization index; AS, Amount solubilized; IAA, Indole-3-acetic acid; SP, Siderophore production; HCN, hydrogen cyanide production. All experiments were performed in triplicates.

### Experimental Designs and Monitoring Post Harvesting of Samples

**Figure 1:**
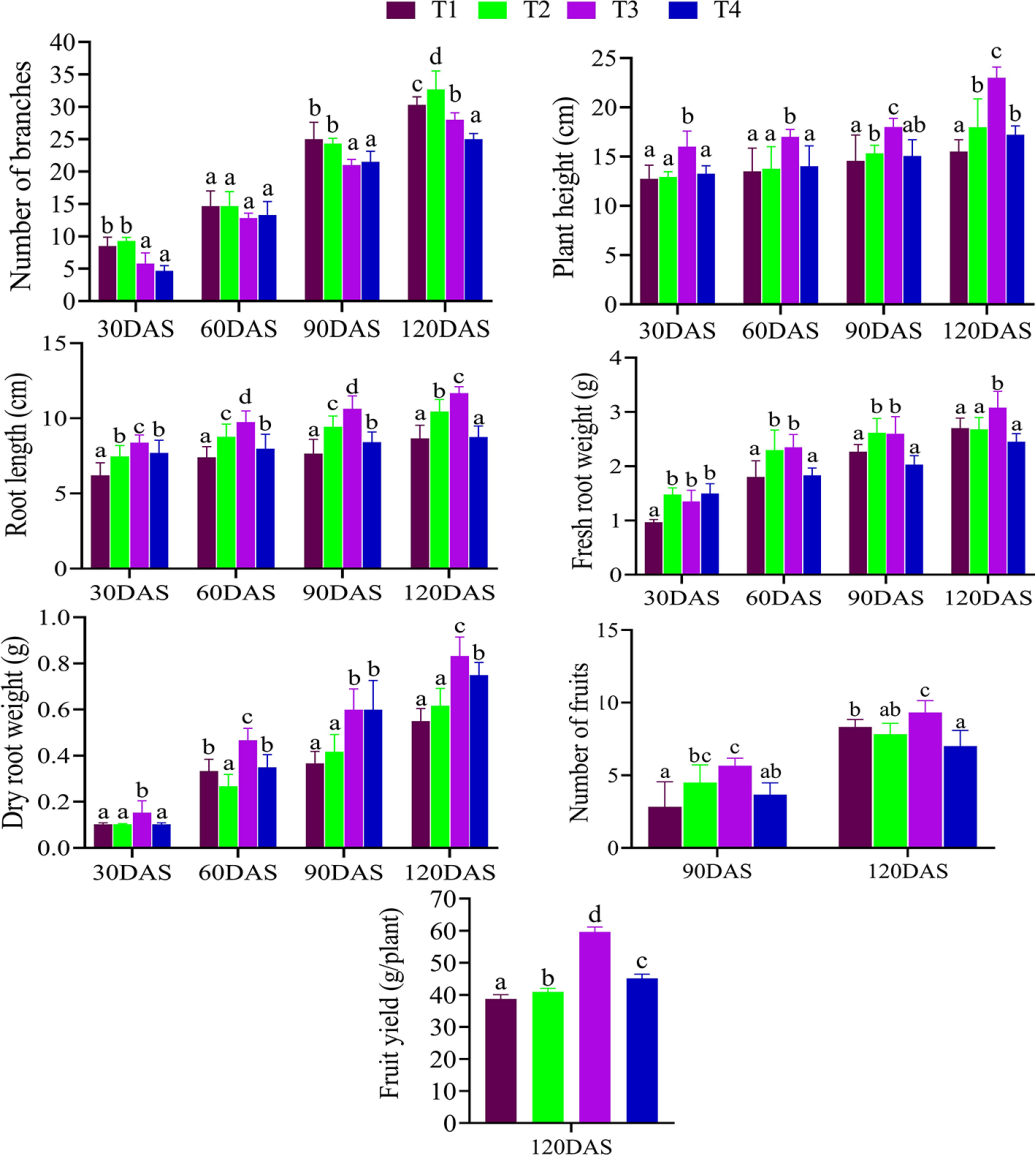
Effects of different bacterial treatment on the various plant growth parameters of *C. annuum* L. at different time intervals (30 DAS, 60 DAS, 90 DAS, and 120 DAS). Where, T1: Untreated control; T2: seeds + CDK15; T3: seeds + CDK25; T4: seeds + Consortium (CDK15 + CDK25); DAS: days after sowing. All the values are in mean ± SD; (n = 6), Level of significance: p < 0.05; Values with different superscripts (a–d) are significantly different (One-way ANOVA followed by Duncan’s multiple range tests).

Healthy seeds of *C. annuum* L. provided by the G. B. Pantnagar University, Uttarakhand, India were bacterized by the selected isolates (CDK15 and CDK25) according to Weller and Cook, (1983). The experiment was laid into four treatment combinations, namely, T1 (seeds: control), T2 (seeds + CDK15), T3 (seeds + CDK25), T4 (seeds + consortium: CDK15 + CDK25). The study was carried out with factorial randomized block design (RBD) at Dehradun, Uttarakhand, India, during the late winters–early summers (January to April) of 2017 and 2018. A pot experiment was conducted to estimate the effect of the bacterial treatments on various planting value (vegetative and reproductive) parameters *viz*., number of branches, plant height, root length, fresh root weight, dry root weight, number of fruits, and fruit yield of *C. annuum* L. at different time intervals (**Figure 1**). For harvesting session, fresh and healthy fruits from each treatment were randomly obtained after 120 days of sowing and transported to the departmental laboratory for proximate and nutrient content analysis. All the samples were washed, shade air-dried followed by pulverization to powder form *via* blender. The pulverized form of the samples was retained at room temperature in air-tight vessels for analysis of proximate chemical contents *viz*., moisture, crude protein, crude fiber, ash, fat, and carbohydrate and mineral contents *viz*., calcium, magnesium, phosphorus, potassium, copper, zinc, and iron of *C. annuum* L.

### Soil Nutrient Analysis

Soil samples were randomly collected at a depth of 10–15 cm from each experimental designed treatment in four sets with triplicate at respective time intervals: 0 days (before sowing), followed by 30 days, 60 days, 90 days, and 120 days after sowing. All the samples collected from various treatments were thoroughly mixed. Before the physico-chemical analysis, all the samples were shade air-dried and passed over a 2-mm sieve. The physico-chemical characteristics *viz*., pH, electrical conductivity, organic carbon, phosphorus, potassium, zinc, magnesium, sulfur, iron, manganese, boron, and copper contents were observed by following the standard procedures (Anderson and Ingram, 1989).

### Proximate Complete Analysis

The proximate complete analysis was carried out to evaluate moisture, crude fiber, crude protein, ash, and fat content of fruit samples by following the standard procedures of the Association of Official Analytical Chemists method (AOAC, 2016). The moisture content of the fruit samples was estimated by the hot air oven method (105°C). Crude protein content was determined in Kjeldhal digestor and automatic distillation system by following the Macro Kjeldahl method. The crude fiber was assessed by complete extraction of the sample *via* a solution of H_2_SO_4_ acid (1.25%) and NaOH solution (1.25%) after the filtrate was ashed; thus, a loss in weight was recorded as crude fiber. For fat content estimation extraction of the sample (2 g) with petroleum ether (boiling point of 40°C to 60°C) was carried out by the soxhlet extraction method. Likewise, 2 g of the samples was weighed in a tarred porcelain crucible for ash content determination followed by incineration at 550°C in an ash muffle furnace. While, the total carbohydrate content was determined by the following formula: Carbohydrate (%) = 100 − (% Moisture + % Crude protein + % Crude fiber + % Fat + %Ash).

### Nutrient Content Analysis

Nutrient content analysis was carried out to measure the macro and micronutrient by following the standard methodology of the Association of Official analytical chemist (AOAC, 2016). Samples harvested from each treatment were placed in the digestion tube, to which 1 ml of conc. H_2_SO_4_ was added. The mixture was placed in a sand heater for 15–20 min, by which the mixture appeared to be of dark color. The mixture was thoroughly cooled and digested in diacid (HNO_3_–H_2_O_2_ acid mixture) for 30–45 min till the color of the mixture became transparent. 100 ml distilled water was added to the mixture to make up the final volume. From the mixture, the macronutrients *viz*., calcium and magnesium, were determined by atomic absorption spectroscopy (Varian AA240) (Mertens, 2005). The micronutrients *viz*., zinc, copper, and iron were also determined by atomic absorption spectroscopy. Similarly, the potassium and phosphorus contents were recorded by flame photometer and UV-VIS spectrophotometer (Shimadzu-64) respectively (Oyeleke, 1984; Ogunlade et al., 2006). The preparation of suitable salts of the nutrients was used as standards.

### Statistical Analysis

The data obtained from the plant growth parameters, soil nutrient analysis (pre and post sowing), proximate complete analysis, and nutrient analysis were examined by Analysis of variance (One-way ANOVA) followed by Duncan’s multiple range test (DMRT) at p ≤ 0.05 level of significance in the IBM-SPSS software (version 25, New York, USA). The principal component analysis (PCA) carried out for determining the statistical correlation between the effect of different bacterial treatments on proximate and nutrient constituents of *C. annuum* L. was conducted in XLSTAT software (France). A Scatter matrix plot with histogram was generated for evaluating the variables’ correlation, thereby signifying pair-wise scatter plots of the variables in a matrix format (Origin software version 9, USA). All figures, volcano plot, and a heat map were generated in Graphpad Prism (version 8, California, USA).

## Results and Discussion

### Identification and Characterization of Bacterial Strains

Both the bacterial strains were characterized physio-morphologically, followed by molecular identification by 16S rRNA gene sequencing. Based on the physio-morphological and biochemical test, both the bacterial strains CDK15 and CDK25 were found to belong to *Bacillus* spp. The physio-morphological and biochemical analysis revealed CDK15 to be Gram-positive, rod-shaped, moderate, cream-colored, and endospore-forming bacterium. It gave positive results for catalase, nitrate reduction, malonate, starch, and gelatin hydrolysis. Whereas, it tests negative for indole, Voges-Proskauer, methyl red, H_2_S production, and citrate utilization. Likewise, CDK25 was Gram-positive, rod-shaped, large, cream-colored, and endospore-forming bacterium. It tests positive for catalase, nitrate reduction, malonate, methyl red, starch, and gelatin hydrolysis. It gave a negative test for indole, Voges-Proskauer, H_2_S production, and citrate utilization. However, the nutrient solubilization and plant growth-promotion analysis revealed CDK25 to be most potent in terms of assays *viz*., phosphate solubilization (281.59 μg/ml), zinc solubilization (20.0 ppm), indole-3-acetic acid production (13.8 μg/ml), hydrogen cyanide production, and siderophore production. Similarly, CDK15 solubilizes phosphate (264.04 μg/ml) and zinc (14.0 ppm) along with the indole-3-acetic acid production (11.6 μg/ml), hydrogen cyanide production, and siderophore production. The molecular characterization results revealed strains CDK15 and CDK25 to be *Bacillus thuringiensis* (MT705245) and *Bacillus megaterium* (MG774438), respectively (**Supplementary Figure S1**). Between both, the strains CDK25 showed remarkable results. Both the strains were maintained in LB medium with 15% glycerol. However, before any experiment, a single bacterial colony was obtained from a stock culture and transferred to a flask containing LB broth followed by incubation on a rotating shaker for 24–48 h at 30 ± 1°C.

### Soil Nutrient Analysis

**Figure 2:**
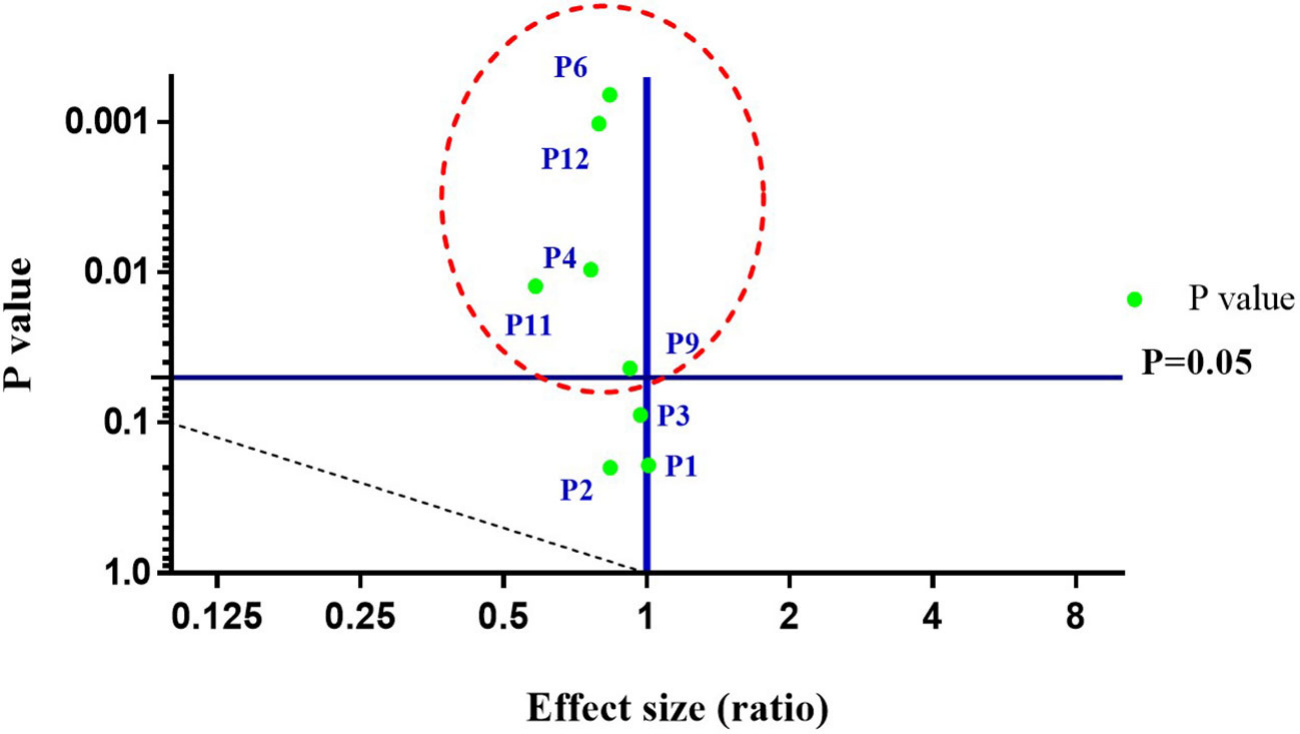
A volcano plot representing effects of bacterial treatments on soil parameters before (0 DAS) and after harvesting (120 DAS) of *C. annuum* L. Where DAS: days after sowing; P1: pH; P2: Electric conductivity; P3: Organic carbon; P4: Phosphorus; P6: Zinc; P9: Iron; P11: Boron; P12: Copper. The parameters *viz*., P4, P6, P9, P11, and P12 represent strong statistical differences (p < 0.05). All the values are arithmetic mean ± SD; n = 6.

The physico-chemical analysis of soil reveals all the parameters in the range: pH (7.25–7.34), electric conductivity (0.15–0.17 ds/m), organic carbon (1.12–1.14%), potassium (53.76–71.68 mg/kg), phosphorus (172.66–548.4 mg/kg), zinc (4.10–4.91 ppm), magnesium (6.90–17.34 ppm), sulphur (22.89–74.10 ppm), iron (19.18–20.86 ppm), manganese (6.06–17.28 ppm), boron (0.46–0.77 ppm), and copper (2.16–2.72 ppm). Results of soil nutrient analysis of all parameters, before and after days of sowing (DAS) varied in the order: 120 DAS > 90 DAS > 60 DAS > 30 DAS > 0 DAS irrespective of pH and electric conductivity (**Table 2**). Further, to establish the relationship between the respective soil parameters precisely before (0 DAS) and after harvesting (120 DAS) of the host plant, a volcano plot was constructed, revealing a correlation between a p-value of statistical test and magnitude of change (fold change) of parameters. The volcano plot depicts, among all parameters, P1 (pH), P2 (electric conductivity), P3 (organic carbon), P4 (phosphorus), P6 (zinc), P9 (iron), P11 (boron), and P12 (copper) to be statistically significant. However, the parameters *viz*., P4, P6, P9, P11, and P12 represent strong statistical differences at (p < 0.05). Thus, the volcano plot illustrates the statistical strength of soil physico-chemical data, thereby exemplifying the levels of significance (**Figure 2**). So, the enrichment in existing soil nutrients may be because of the efficacy of plant growth-promoting bacteria. However, the existing literature also proposes the starring role of PGPB in having nutrient solubilization ability for enriching the nutritional status of soil and host plant (Rokhzadi and Toashih, 2011; Shen et al., 2011). Furthermore, several studies also support the efficiency of PGPB in boosting the soil nutrient status, besides elevating the plant biological and nutritional traits (Para-Cota Fannie et al., 2014; Das and Singh, 2014; Pandey et al., 2018a). Thus, the analysis demonstrates the effectiveness of plant growth-promoting bacteria in transforming complex fractions of soil nutrients (organic and inorganic) to bio-available fractions *via* various plant growth-promoting attributes, thereby altering soil nutrients proportions.

**Table 2:** Soil nutrient analysis at different time intervals (0 DAS, 30 DAS, 60 DAS, 90 DAS, and 120 DAS).

**Parameters**	**Units**	**Days after sowing (DAS)**				
		0 DAS	30 DAS	60 DAS	90 DAS	120 DAS
pH	–	7.34 ± 0.01^e^	7.30 ± 0.005^d^	7.27 ± 0.01^abc^	7.25 ± 0.01^c^	7.26 ± 0.03^e^
Electric conductivity	(ds/m)	0.16 ± 0.02^a^	0.15 ± 0.005^a^	0.16 ± 0.01^a^	0.17 ± 0.01^a^	0.17 ± 0.01^a^
Organic carbon	(%)	1.12 ± 0.01^a^	1.12 ± 0.005^ab^	1.13 ± 0.005^a^	1.14 ± 0.005^a^	1.14 ± 0.015^b^
Phosphorus	(mg/kg)	53.76 ± 1.70^h^	57.82 ± 0.3^g^	61.14 ± 0.1^f^	65.37 ± 0.8^g^	71.68 ± 0.9^h^
Potassium	(mg/kg)	172.66 ± 1.06^i^	197.23 ± 2.6^h^	287.2 ± 12.7^g^	415.2 ± 5.6^h^	548.4 ± 1.06^j^
Zinc	(ppm)	4.10 ± 0.005^c^	4.20 ± 0.02^c^	4.51 ± 0.04^ab^	4.71 ± 0.02b^c^	4.91 ± 0.02^a^
Magnesium	(ppm)	6.90 ± 0.03^de^	8.25 ± 0.03^d^	12.03 ± 0.06^cd^	14.33 ± 0.04^d^	17.34 ± 0.07^f^
Sulfur	(ppm)	22.89 ± 0.05^g^	27.33 ± 0.42^f^	49.78 ± 0.40^e^	61.51 ± 0.43^f^	74.10 ± 0.01^i^
Iron	(ppm)	19.18 ± 0.06^f^	19.37 ± 0.02^e^	19.61 ± 5.7^d^	19.88 ± 0.02^e^	20.86 ± 0.35^g^
Manganese	(ppm)	6.06 ± 0.05^d^	8.37 ± 0.04^d^	10.27 ± 0.01^bcd^	14.84 ± 0.07^d^	17.28 ± 0.06^f^
Boron	(ppm)	0.46 ± 0.03^a^	0.51 ± 0.02^a^	0.63 ± 0.03^a^	0.67 ± 0.02^a^	0.77 ± 0.02^ab^
Copper	(ppm)	2.16 ± 0.01^b^	2.21 ± 0.01^b^	2.41 ± 0.03^a^	2.51 ± 0.01^ab^	2.72 ± 0.02^c^

Where, DAS, Days of sowing; All values are arithmetic mean ± SD (n = 6), with different letters (a–j) in the same row are significantly different (p < 0.05) determined by One-way ANOVA followed by Duncan’s Multiple Range Test.

### Effects of Bacterial Treatments on Proximate Constituents

**Figure 3:**
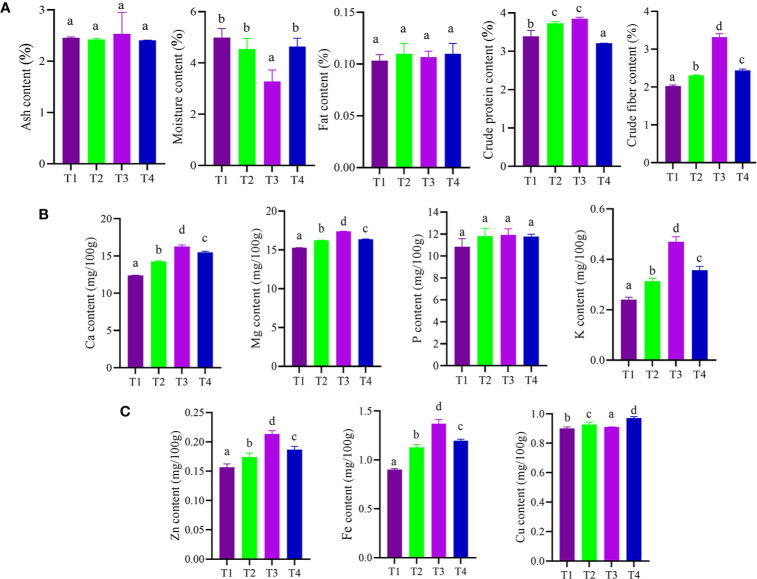
**(A)** Effect of bacterial treatment on proximate constituents. **(B)** Effect of bacterial treatment on macronutrient contents. **(C)** Effect of bacterial treatment on micronutrient contents of *C. annuum* L. Where M: Moisture; CP: Crude protein; CF: Crude fiber; A: Ash; F: Fat; K: Potassium; Ca: Calcium; Mg: Magnesium; P: Phosphorus; Zn: Zinc; Fe: Iron; Cu: Copper; T1: Untreated control; T2: seeds + CDK15; T3: seeds + CDK25; T4: seeds + Consortium (CDK15 + CDK25). Values are expressed as mean ± SD (n = 6), Level of significance: p < 0.05; Values with different superscripts are significantly different (One-way ANOVA followed by Duncan’s multiple range tests).

The results of different treatments on the proximate chemical composition of dried pulverized fruit samples of *C. annuum* L. reveals that treatment (T3) with CDK25 bacterial strain showed significant moisture content 3.28% followed by treatment (T2) with CDK15 bacterial strain (4.54%) over the control (T1) (**Figure 3A**). Since the shelf life of spices is significantly influenced by its moisture content and is frequently an index of quality (Bradley, 2010), so, a high level of moisture could spoil the *C. annuum* L. samples. Thus, the analysis goes well with the fact that less the moisture content the more is the shelf life, indicating less microbial activity, ultimately leading to prolonged food storage life (Dashak et al., 2001). Similar to this, the protein content analysis result revealed that all the treatments ranged between 3.20 and 3.84%, where significant increment in the amount of crude protein (3.84%) was recorded by the samples obtained from T3 treatment (seeds + CDK25) followed by treatment T2 (seeds + CDK15) over the control (T1). The maximum content of crude protein in *C. annuum* L. may also be owing to the existence of active proteinous metabolite, like capsaicin. Our study goes well with the report of Kang et al. (2012), demonstrating the enhancement of protein content of cucumber by application of PGPRs. Similar to protein, fibers are one of the food constituents crucial for human health. The present analysis results showed that the highest value of 3.31% crude fiber was recorded in treatment T3 (seeds + CDK25) followed by 2.44% in treatment T4 [seeds + Consortium (CDK15 + CDK25)] as compared to control (T1). Likewise, ash content analysis results revealed treatment T3 to be highest among all with an amount of 2.53% followed by control (T1) with an amount of 2.45%. High ash contents suggest more availability of minerals in the samples, which is further confirmed by the significant increment in calcium, magnesium, phosphorus, potassium, copper, zinc, and iron content (**Figures 3B, C****)**. However, a substantial increase in fat content (0.11%) was recorded in treatments T2 and T4, treatment with individual bacteria CDK15 and a combination of two bacteria (CDK15 + CDK25), respectively. High levels of carbohydrate were found in all treatments with a slight difference. However, treatment T1 (87%) and T4 (87.1%) showed remarkably high carbohydrate value followed by treatment T3 (86.9%) and T2 (86.8%). However, even if carbohydrates are present in high value they might not be nutritionally quantifiable because maximum of them are generally bound to remain undigested in the body (Gloria et al., 2010). So, both the advantageous plant growth-promoting bacterial strains (CK25 followed by CDK15) were proficient enough to sustain ample amounts of proximate chemical components. Similar other studies also indicated the enhancement of carbohydrate, protein, and proximate content of cauliflower, chilli, brinjal, wheat, and amaranth by plant growth-promoting bacterial treatment (Kalita et al., 2015; Al-Erwy et al., 2016; Pandey et al., 2018b).

### Effects of Bacterial Treatments on Nutrient Constituents

**Figure 4:**
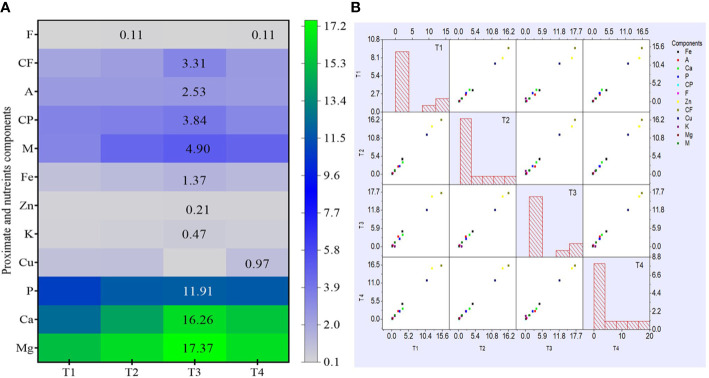
**(A)** Heat map analysis of proximate and nutrient constituents. **(B)** A Scatter Matrix plot with histogram demonstrating pair-wise scatter plots of variables in a matrix format, determining variables correlation. Where, M: Moisture; CP: Crude protein; CF: Crude fiber; A: Ash; F: Fat; K: Potassium; Ca: Calcium; Mg: Magnesium; P: Phosphorus; Zn: Zinc; Fe: Iron; Cu: Copper; T1: Untreated control; T2: seeds + CDK15; T3: seeds + CDK25; T4: seeds + Consortium (CDK15 + CDK25). Values are expressed as mean ± SD (n = 6); Level of significance: p < 0.05.

Similar to complete proximate analysis, the nutrient (macro and micro) analysis of dried pulverized fruit samples of *C. annuum* L. collected from different treatments was also evaluated. The analysis results reveal that treatment with bacterial isolate CDK25 (T3) generally enhanced the entire nutrient component (**Figures 3B, C****)** where the maximum calcium content was recorded by treatment T3 (seeds + CDK25) with a value of 16.26 mg/100 g followed by T1 (control) with a value of 15.49 mg/100 g. As, calcium is supplemented in a small portion of the total diet but is also responsible for several metabolic processes, muscle contraction, and blood coagulation (Martınez et al., 1999). Moreover, calcium in conjugation with manganese, phosphorous, magnesium, ascorbic acid, vitamin A, D, protein, and chlorine is involved in the formation of bone. Likewise, the highest magnesium content (17.37 mg/100 g) was recorded by treatment T3 (seeds + CDK25) followed by treatment T4: a combination of bacteria (CDK15 + CDK25) with a value of 16.35 mg/100 g. Maximum phosphorous content (11.91 mg/100 g) was also recorded by treatment T (seeds + CDK25) while minimum content by control (T1) with a value of (10.84 mg/100 g). Similarly, the maximum potassium content (0.47 mg/100 g) occurred due to T3: treatment with individual bacteria CDK25 followed by T4 (0.35 mg/100 g) combination of bacteria (CDK15 + CDK25). The earlier report supports our study, stating the application of plant growth-promoting bacteria in an increment of phosphorous and potassium content in corn (Adesemoye et al., 2008), phosphorous content in soybean (Egamberdieva et al., 2016). Besides this, the literature also reveals the application of nutrient solubilizing plant growth-promoting bacteria (PGPBs) for uplifting plant nutrient contents (Rokhzadi and Toashih, 2011). Similar to this macronutrient, the micronutrient content of *C. annuum* L. fruit samples was also evaluated. As zinc plays a crucial role in more than fifty enzymes, it also has a significant role in cell growth, cell division, wound healing along with the breakdown of carbohydrates (Gloria et al., 2010). In the micronutrient analysis, the highest zinc content was observed in treatment T3 (seeds + CDK25) with a value of 0.21 mg/100 g followed by T2 (seeds + CDK15) with a value of 0.17 mg/100 g. Likewise, one of the micronutrients is iron, an abundant element on earth, indispensable for every living being. It plays numerous functions in the body *viz*., oxygen-binding hemoglobin, as a catalytic center in many enzymes (cytochrome). The highest iron content (1.37 mg/100 g) was found to be maximum in treatment T3 (seeds + CDK25) followed by treatment T4 (seed + consortium: CDK15+CDK25) and T2 (seeds + CDK15) with a value of 1.19 mg/100 g and 1.13 mg/100 g respectively. However, copper content data depicts all treatment to be significant. Both the constituent (proximate and nutrient) datasets were also visually analyzed by heat map and scatter matrix plot, representing variation in each constituent caused by different bacterial treatments *via* variation in color and their correlation, respectively (**Figures 4A, B****)**. PGPBs are recognized well for possessing PGP assets subsequently solubilizing and transforming several soil nutrients in their vicinity, thereby facilitating plant nutrient supplies. So, the enrichment in the nutrient components could be because of the PGP activities of the beneficial bacilli strains.

### Principal Component Analysis of Proximate and Nutrient Constituents

**Figure 5:**
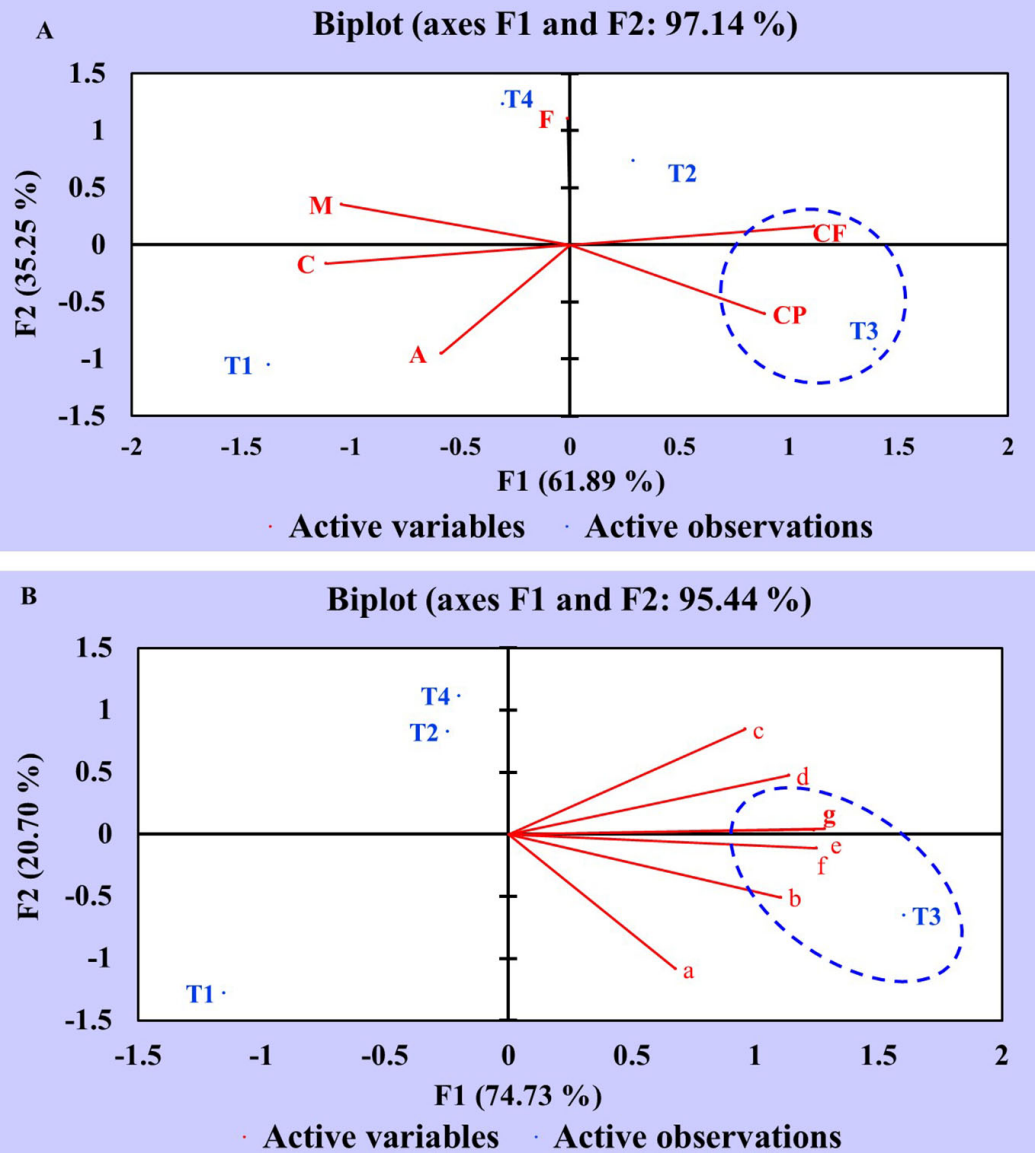
Principal Component Analysis showing the correlation between the effect of different treatments and variables of *C. annuum* L. **(A)** Principal component analysis of different treatments and proximate chemical constituent variables. **(B)** Principal component analysis of different treatments and nutrient constituent variables. Where, treatments: T1: Untreated control; T2: seeds + CDK15; T3: seeds + CDK25; T4: seeds + Consortium (CDK15 + CDK25); M: Moisture, CP: Protein, CF: Crude Fiber, A: Ash, F: Fat, C: Carbohydrate, a: Calcium; b: Magnesium; c: Phosphorous; d: Potassium; e: Copper; f: Zinc; g: Iron.

Principal component analysis (PCA) of proximate and nutrient (macro and micro) constituents was executed to assess the statistical correlation between different treatments (T1, T2, T3, T4) and variables. The data obtained were statistically analyzed using XLSTAT software. The PCA of proximate chemical constituents is elucidated in a Biplot graph (F1 and F2 axes) with two components: component 1 (F1:61.89%) and component 2 (F2:35.25%) (**Figure 5A**). The analysis results revealed that treatment T3 (seeds + CDK25) showed a potential influence on the variables *viz*., crude protein (CP) and crude fiber (CF) as manifested by positive correlation in addition to the existence of T3, CP, and CF in the same domain. However, treatment T2 (seeds + CDK15) was also present in the positive domain but did not correlate with any of the constituents, thus showing a minor effect. On the contrary, the variables *viz*., T1 (uninoculated control), T4 (seed + consortium: CDK15 + CDK25), A (Ash), C (Carbohydrate), and M (Moisture) appeared to be in the negative domain, concluding them to be least responsible in terms of improving proximate chemical constituents of test crops. The fat content was not correlated with any of the treatments as it lies on the axis.

Similar to the proximate chemical constituents, nutrient constituent data was also statistically analyzed by PCA. The PCA of diverse variables and treatments is explained in a Biplot graph (F1 and F2 axes) with two components: component 1 (F1:74.73%) and component 2 (F2:20.70%) as displayed in **Figure 5B**. The data depicts an increment of the nutrient (macro and micro) contents *viz*., magnesium (b), potassium (d), copper (e), iron (g), and zinc (f) which occurred due to T3 (seeds + CDK25) as evidenced by the appearance in the same domain, representing a positive correlation. However, the minor impact was observed in treatment T1 (uninoculated control) as evidenced by the appearance in the negative domain, revealing less effectiveness in enhancing the nutrient contents. Perhaps, this may be due to the seeds being deprived of PGP bacterial coating in treatment T1 (uninoculated control). Thus, the PCA analysis data depicts the positive correlation between the different treatments with the variables, which validate the increase in constituents by the bacterial treatments, where the analysis data favored T3 to be the best treatment for obtaining the maximum proximate and mineral components. So, the “*B. megaterium*” strain CDK25 was proficient in elevating the complete nutritional status of *C. annuum* L., besides augmenting the vegetative and reproductive states.

Hence, the application of the promising PGP bacterial strain can increase the proximate and mineral content in the plant besides enhancing plant growth parameters and soil fertility, as the positive correlation between various treatments and variables approves the statement. This is possible because PGPB increases the accessibility of soluble nutrients in the soil, which is further utilized by the plant.

## Conclusion

To date, this is the first report bestowing concurrent potential of cow dung bacterium “*B. megaterium”* in enriching the chemical proximate and mineral composition of *C. annuum* L, besides stimulating vegetative and biological growth parameters. The nutritional value obtained was statistically evaluated through principal component analysis, thereby supporting the current study results. The soil nutrient analysis at different intervals of time was also assessed statistically by constructing a volcano plot graph revealing substantial statistical differences before and after sowing, concluding enhancement in soil mineral transformation, thus augmenting crop chemical and nutritive value. Therefore, by the use of this eco-friendly, cheap, and easily accessible bacterium, farmers can be encouraged toward sustainable agriculture, thereby enhancing the nutritive and commercial value of the economically valued crop. Moreover, the study also proposes the practice of using beneficial bacteria for the enduring nutrients’ discharge, henceforth contributing their potential in nourishing the fitness of plant and soil network.

## Data Availability Statement

The datasets presented in this study can be found in online repositories. The names of the repository/repositories and accession number(s) can be found below: MG774438, MT705245.

## Author Contributions

DKM designed and supervised the experiments. KB performed the experiments and prepared the manuscript. All authors contributed to the article and approved the submitted version.

## Conflict of Interest

The authors declare that the research was conducted in the absence of any commercial or financial relationships that could be construed as a potential conflict of interest.

## Supplementary Material

Figure S1 Phylogenetic analysis of two selected plant growth-promoting isolates CDK15 and CDK25 based on 16S rRNA gene sequencing.
