# Supplementary figures and images for: Bacillus megaterium Strain CDK25, a Novel Plant Growth Promoting Bacterium Enhances Proximate Chemical and Nutritional Composition of Capsicum annuum L
Source: Front Plant Sci. 2020 Jul 30;11:1147. doi: 10.3389/fpls.2020.01147 (PMC7406793; doi:10.3389/fpls.2020.01147)

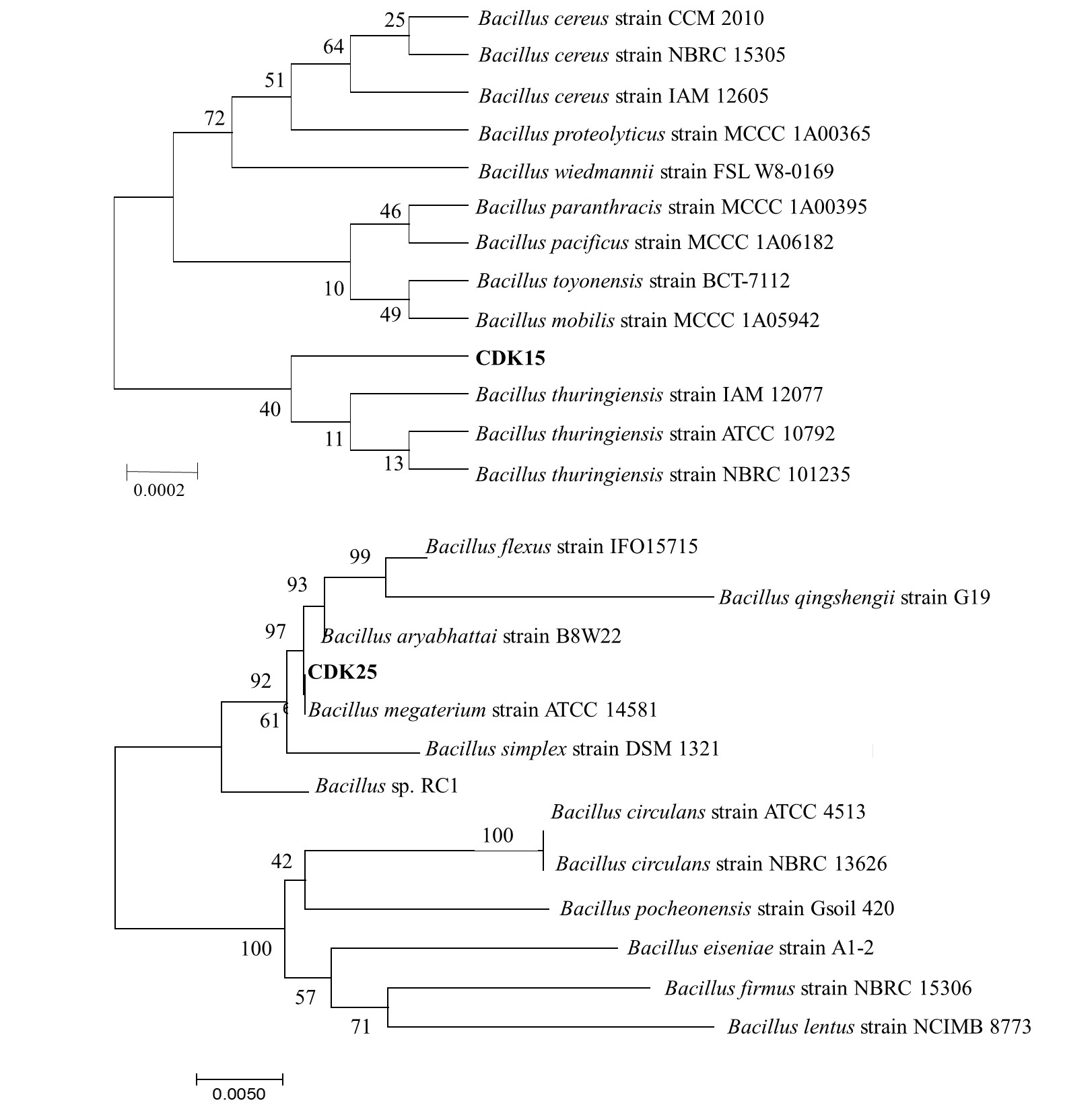

Supplement: Figure S1 — Phylogenetic analysis of two selected plant growth-promoting isolates CDK15 and CDK25 based on 16S rRNA gene sequencing. [file Image_1.jpeg]
